# Aerosol-Assisted Extraction of Silicon Nanoparticles from Wafer Slicing Waste for Lithium Ion Batteries

**DOI:** 10.1038/srep09431

**Published:** 2015-03-30

**Authors:** Hee Dong Jang, Hyekyoung Kim, Hankwon Chang, Jiwoong Kim, Kee Min Roh, Ji-Hyuk Choi, Bong-Gyoo Cho, Eunjun Park, Hansu Kim, Jiayan Luo, Jiaxing Huang

**Affiliations:** 1 Rare Metals Research Center, Korea Institute of Geoscience & Mineral Resources, Deajeon. 305-350, Korea; 2 Department of Nanomaterials Science and Engineering, University of Science & Technology, Deajeon. 305-350, Korea; 3 R&D Center for Valuable Recycling, Korea Institute of Geoscience and Mineral Resources, Daejeon. 305-350, Korea; 4 Department of Energy Engineering, Hanyang University, Seoul. 133-791, Korea; 5 School of Chemical Engineering and Technology, Tianjin University, Tianjin 300072, China; 6 Department of Materials Science and Engineering, Northwestern University, Evanston, Illinois 60208, USA

## Abstract

A large amount of silicon debris particles are generated during the slicing of silicon ingots into thin wafers for the fabrication of integrated-circuit chips and solar cells. This results in a significant loss of valuable materials at about 40% of the mass of ingots. In addition, a hazardous silicon sludge waste is produced containing largely debris of silicon, and silicon carbide, which is a common cutting material on the slicing saw. Efforts in material recovery from the sludge and recycling have been largely directed towards converting silicon or silicon carbide into other chemicals. Here, we report an aerosol-assisted method to extract silicon nanoparticles from such sludge wastes and their use in lithium ion battery applications. Using an ultrasonic spray-drying method, silicon nanoparticles can be directly recovered from the mixture with high efficiency and high purity for making lithium ion battery anode. The work here demonstrated a relatively low cost approach to turn wafer slicing wastes into much higher value-added materials for energy applications, which also helps to increase the sustainability of semiconductor material and device manufacturing.

Silicon (Si) is the most widely used material in the semiconductor and photovoltaic industries^1^,^2^,^3^. However, a large amount of silicon material in the form of debris is generated during the slicing of silicon ingots into thin wafers for the fabrication of integrated-circuit chips and solar cells. Approximately 40% of a silicon ingot is lost as saw dust in the form of nano-sized silicon particles less than 200 nm in diameter^4^,^5^. Unfortunately, this large amount of silicon sludge falls into disuse as waste, causing a severe loss of valuable resources as well as environmental contamination. Recently, recovery of these silicon particles from the sludge wastes has been of great interest due to the demand for the recycling of valuable high-purity silicon and environmental protection. Typically silicon has been recovered from the sludge waste diluted in solvent through centrifugation- or filtration-based methods, and converted into other silicon containing compounds such as Si_3_N_4_, SiCl_4_ and SiC, etc^3^,^4^,^6^,^7^,^8^,^9^,^10^,^11^,^12^ with the purpose of creating higher value added materials. It would be highly desirable if one can directly repurpose the recovered Si particles for high value applications themselves. Here we demonstrate one such application, where recovered Si debris particles are directly used as the anode materials for lithium ion batteries. In this study, we present a novel one-step separation process, which extracts Si nanoparticles from a mixture of Si and SiC particles through the use of an ultrasonically driven aerosol spray-drying method. In contrast to centrifuge or filtration based methods, the aerosol process is rapid, simple, eco-friendly, and can produce dried Si nanoparticles that are ready to be used for making the anode of lithium-ion batteries. Using such recycled Si nanoparticles for battery applications is a relatively low cost approach to convert a waste into new materials with significantly enhanced values, which also helps to increase the sustainability of semiconductor manufacturing.

## Results

**Figure 1:**
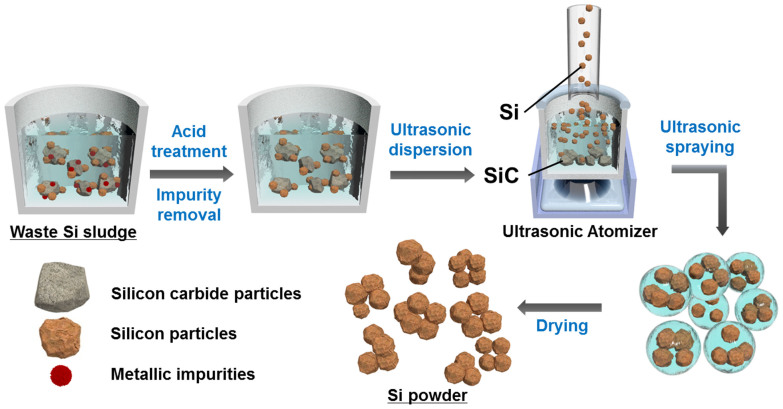
Schematic drawings illustrating the ultrasonic aerosol assisted Si extraction process.

**Figure 2:**
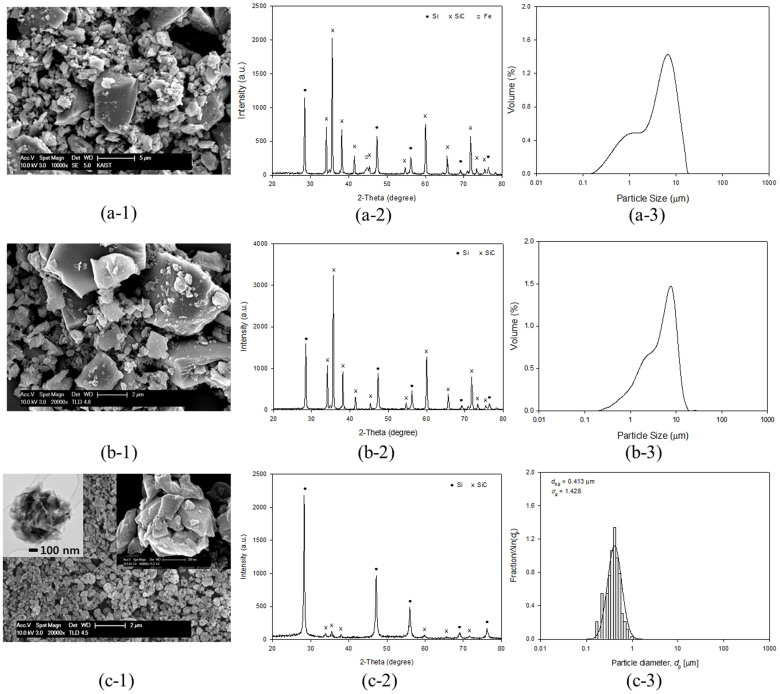
SEM images (a-1, b-1, c-1) and corresponding XRD patterns (a-2, b-2, c-2) and particle size distribution (a-3, b-3, c-3) of the starting (1) sludge waste powders, (2) the powders after acid treatment and (3) the Si product obtained by aerosol extraction, respectively.

**Figure 3:**
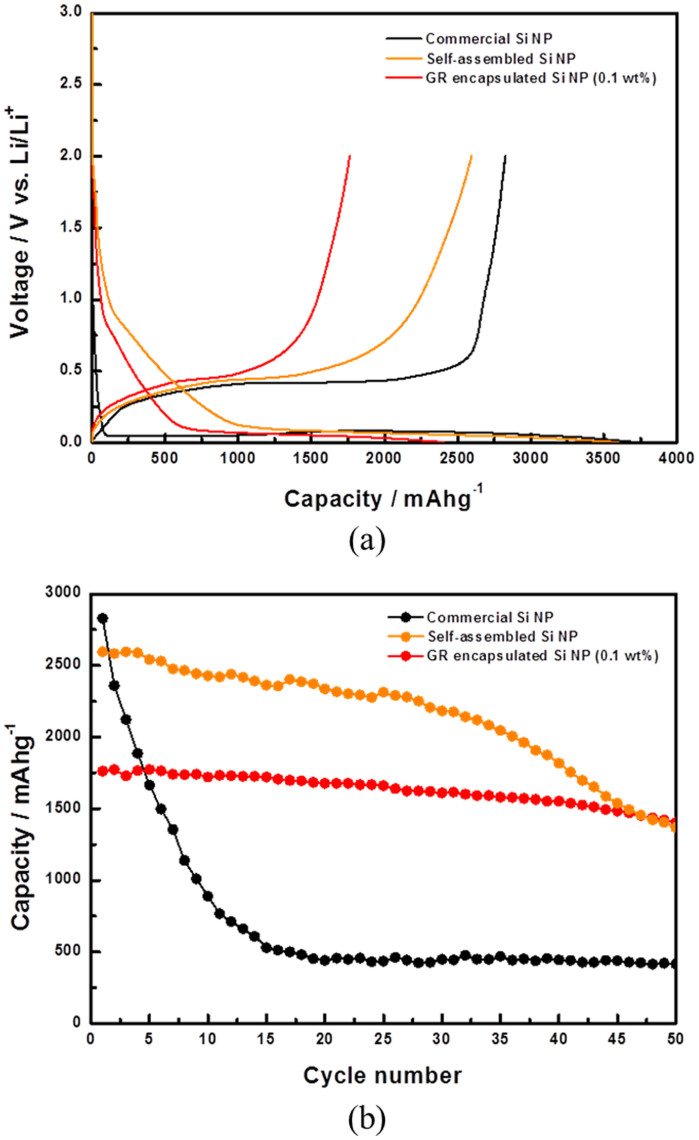
(a) Voltage profiles and (b) capacity retention of aerosol extracted Si nanoparticle agglomerates, GR-encapsulated Si nanoparticles and commercially available Si nanoparticles.

Figure 1 shows schematic illustration of the aerosol-assisted Si recovery process. First, dried sludge containing a mixture of Si and SiC was redispersed in water and subject to ultrasonic atomization to disperse the particles and nebulize water droplets at the same time. Since the SiC particles have much larger size, higher density and much higher hardness, they can effectively grind the Si debris during ultrasonication, resulting in smaller and lighter Si particles that can also better disperse in water. Therefore, when the nebulized droplets leave water surface, Si particles are more likely to be captured and depart from the solution. As the carrier gas (Ar) is introduced into the reservoir, the droplets fly through a pre-heated tube furnace. During the flight, capillary force generated from solvent evaporation rapidly assembles the Si nanoparticles into clusters of submicron diameters. The dried product is then collected in a filter under vacuum, and analyzed with scanning electron microscopy (SEM) and powder X-ray diffraction (XRD). The particle size distribution was determined by a particle size analyzer (PSA) and counting more than 200 particles from the SEM images of corresponding samples as well. As shown in Figures 2a-1 and 2a-3, particles in the dried sludge have diverse size distribution from 0.2 to 15 μm in diameter. Most of the larger particles are SiC while the smaller ones are composed of mainly Si with a minor portion of SiC. The XRD result shows that the sludge consists of mainly Si and SiC and a small amount of Fe impurity (Fig. 2a-2). The Fe impurities can be easily removed by acid treatment as indicated by the XRD pattern (Fig. 2b-2)^9^. The SEM images and the size distribution of the sludge after acid treatment show that the initial particle morphology and size did not change greatly compared to those of the as-dried sludge (Figs. 2b-1 and 2b-3). Figure 2c-1 shows SEM and TEM images of the Si nanoparticle clusters obtained after aerosol extraction. SEM and TEM observations (Fig. 2c-1) show that the as-recovered particles are uniformly sized agglomerates of nanosized particles, resulting from the self-assembly of small nanoparticles due to the capillary force which arises during the evaporation of the solvent in the sprayed droplets. It was found that 80 wt% of Si was recovered from the silicon sludge by the aerosol process. Corresponding XRD pattern indicates the recovered material was mainly composed of Si with much reduced content of SiC (Fig. 2c-2). The mass fraction of SiC in the recovered material was measured to be 3.8 wt%, according to a chemical measurement method^4^. The sample shown in Figure 2 was collected from a sludge suspension with starting solid concentration of 0.5 wt%. The particle size of the as-prepared Si agglomerates ranged from 0.1 to 1.5 μm and centered at around 0.4 μm (Fig. 2c-3). With higher initial solid concentration, larger agglomerates can be obtained without significantly increasing the amount of SiC in the final product (supplementary materials Figure S1). The SEM image, XRD pattern, and particle size distribution of the residue in the reservoir of ultrasonic atomizer are shown in Figure S2. It is exhibited that the residue was mainly composed of SiC particles ranged from 2 to 20 μm after Si particles were recovered from the Si sludge powder. It is considered that the existence of remaining Si particles in the residue was due to strong agglomeration between Si and SiC particles. These results demonstrate that ultrasonic aerosol spray-drying method is promising process for recovering Si nanoparticles from waste sludge.

Si nanoparticles have attracted significant interest as high performance anode materials for lithium-ion batteries^13^,^14^,^15^,^16^,^17^,^18^,^19^,^20^. Although much higher purity and extensive reprocessing are required for recycling Si for semiconductor applications, the powder form of our recovered Si nanoparticles are very suitable for battery applications. Commercially available Si nanoparticles tend to have much more uniformly distributed sizes and shapes, and thus can achieve higher packing density in solid state. In contrast, our recovered Si nanoparticles are irregular and non-uniform, which leads to more free volume inside the agglomerates obtained after aerosol processing. Moreover, the sub-micron size and near spherical shape of the agglomerates lead to additional porosity at slightly larger length scale upon densification for battery applications. These build-in porosities should facilitate electrolyte transport and could better accommodate volume expansion/contraction during lithiation/delithiation cycles, which should lead to better cycling stability. Figure 3 shows the electrochemical performances of the aerosol recovered Si nanoparticles agglomerates. Compared to commercially available Si nanoparticles with the size of around 100 nm, the recovered silicon nanoparticles agglomerates indeed show superior capacity retention. In an earlier work, we demonstrated that wrapping commercially available Si nanoparticles with crumpled graphene shells^24^ can significantly improve their cycling stability^25^,^26^. As expected, here wrapping the recovered Si noparticles agglomerates with crumpled graphene shells (supplementary materials Figure S2) can also further improve capacitance retention.

## Discussion

We demonstrated an ultrasonic aerosol spray drying route that can directly extract Si nanoparticles from a suspension of wafer slicing sludge waste. Instead of converting Si to other compounds using expensive chemical processing techniques, we show that the as-recovered Si nanoparticles can be directly used for making anodes of lithium ion batteries. Although the covered Si nanoparticles are very non-uniform in size and shape, they are assembled into sub-micron sized, near-spherical agglomerates, which can pack to form a solid anode with ample free volumes at different length scales, leading to exceptional capacity retention than commercially available Si nanoparticles. This is a relatively low cost strategy that can simultaneously recover an expensive material from industrial wastes, repurpose them for much higher value-added applications, and reduce potentially negative environmental impacts.

## Methods

### Materials

The sludge used in this study was from a Korean semiconductor company. It consisted of Si particles less than 200 nm in diameter generated as kerfs, SiC particles of less than 20 microns from the wafer-slicing saw, and metal impurities from the fragments of cutting wire mixed in ethylene glycol based cutting fluid. The sludge was dried 180°C for 10 hrs to yield powders.

### Extraction of silicon

2 g of the dried sludge powders were first washed with HCl to remove metal impurities and then dispersed in 200 mL of distilled water and agitated to obtain a colloidal suspension, which was then nebulized under ultrasonication to separate the silicon nanoparticles from silicon carbide. During sonication, dried argon with a controlled flow rate of 0.5 to 2.0 L/min was introduced into the reservoir of the ultrasonic atomizer (1.7 MHz, UN-511, Alfesa Pharm Co.) to transport of the aerosol droplets to a vertical tube furnace (tube diameter = 1 in) pre-heated at 300 to 500°C. The dried Si particles were collected by a filter at the end of the tube under vacuum.

### Synthesis of GR-encapsulated Si

GO was prepared by a modified Hummers' method and purified according to methods reported previously^21^,^22^. GO (1 mg/mL) and the as-recovered Si (1–5 mg/mL) were mixed in water and nebulized by a commercial mini spray dryer (CMSD, B290, Buchi). The CMSD had a two-fluid nozzle in which the precursor solution and dispersion air were introduced into the inner and outer tubes, respectively^23^. Precursor droplets were formed from a continuous flow of the precursor solution at the nozzle tip. For the CMSD, the solution flow rate and the temperature were fixed at 4.5 mL/min and 190°C, respectively. The product was collected by a cyclone and a bag filter in series at the exhaust, and then further annealed at 800°C in Ar for 2 h.

### Characterization

The particle morphologies and sizes of the as-prepared Si particles and GR-encapsulated Si were characterized using a field emission scanning electron microscope (FE-SEM; FEI, Sirion), a transmission electron microscope (TEM; JEOL, JEM-ARM200F), and a particle size analyzer (PSA, Mastersizer 2000, Malvern). The size distribution of the as-recovered Si particles was also determined from SEM micrographs by counting over 200 particles. The elemental composition of the samples was measured by an inductively coupled plasma mass spectrometer (ICP/MS; PerkinElmer DRCII). The composition and crystallinity of the sludge powders were analyzed by X-ray diffractometry (XRD; Rigaku, RTP 300 RC).

### Electrochemical Test

Charge/discharge tests were done using a CR2032-type coin cell. Metallic lithium was used as the counter electrode. The working electrode was fabricated by initially pasting a mixture of the electrode material, carbon black and polyamide imide as a binder (Solvay) at a weight ratio of 80:10:10 onto a copper foil (12 mm diameter) and compressing this mixture at 10 MPa. The typical mass loading level was about 0.5 mg per square centimeter of the electrode. The electrode was dried at 120°C for 2 h under a vacuum before being assembled into a coin cell in an Ar-filled glove box. The electrolyte solution was 1 M LiPF_6_/ethylene carbonate (EC)/dimethyl carbonate (DMC) (1:1 by volume). A microporous glass-fiber membrane (Whatman) was used as the separator. Galvanostatic charge/discharge measurements were conducted with a TOSCAT3000 (Toyo, Japan) at various current densities with voltages between 0.005 and 3 V vs Li/Li^+^. Lithium insertion into the Si electrode was referred to as the discharge, and extraction was referred to as the charge. The capacity was determined based on the mass of the electrode materials.

## Author Contributions

H.D.J. and J.H. initiated and coordinated the project. H.D.J., H.K., J.L. and J.H. wrote the manuscript. H.K., H.C. and J.K. carried out the experiment to extract the Si particles from waste Si sludge. K.R. and J.C. prepared Figure 1, 2 and supplemental figures. E.P. and Ha.K. measured the electrical performance of the as-prepared Si particles and graphene encapsulated Si for lithium ion battery anode and prepared Figure 3. Ha.K. revised manuscript on the results of the electrical performance of the as-prepared samples. B.C. investigated status of the generation of waste Si sludge and previous Si recycling technology. All authors discussed the results and contributed to the writing of the manuscript.

## Supplementary Material

Supplementary Information Supplementary Information
