# Supplementary material for: Aerosol-Assisted Extraction of Silicon Nanoparticles from Wafer Slicing Waste for Lithium Ion Batteries
Source: Sci Rep. 2015 Mar 30;5:9431. doi: 10.1038/srep09431 (PMC4377548; doi:10.1038/srep09431)
Supplement: Supplementary Information [file srep09431-s1.doc]

Supplementary Information

**Aerosol-Assisted Extraction of Silicon Nanoparticles from Wafer Slicing Waste for**

**Lithium Ion Batteries**

Hee Dong Jang^1,2, †,*^, Hyekyoung Kim^1,2, †^, Hankwon Chang^1,2^, Jiwoong Kim^1,2^, Kee Min Roh^1^, Ji-Hyuk Choi^1^, Bong-Gyoo Cho^3^, Eunjun Park^4^, Hansu Kim^4,**^, Jiayan Luo^5^, Jiaxing Huang^6,***^

^1^Rare Metals Research Center, Korea Institute of Geoscience & Mineral Resources, Deajeon, 305-350, Korea

^2^Department of Nanomaterials Science and Engineering, University of Science & Technology, Deajeon, 305-350, Korea

^3^R&D Center for Valuable Recycling, Korea Institute of Geoscience and Mineral Resources, Daejeon, 305-350, Korea

^4^Department of Energy Engineering, Hanyang University, Seoul, 133-791, Korea

^5^School of Chemical Engineering and Technology, Tianjin University, Tianjin 300072, China

^6^Department of Materials Science and Engineering, Northwestern University, Evanston,

Illinois 60208, USA


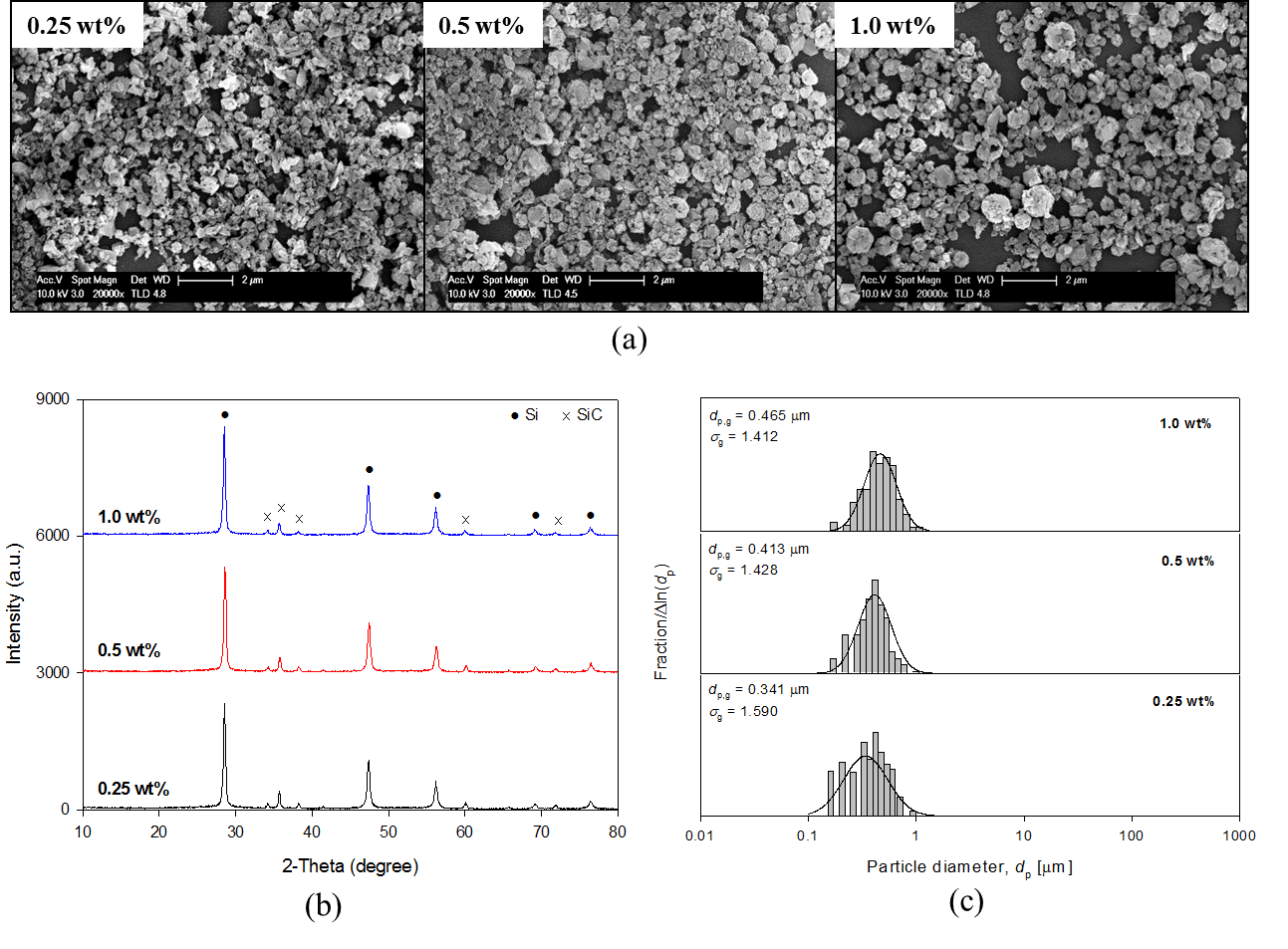


**Figure S1**. (a) SEM images of the as-recovered particles with respect to the initial content of solids in the sludge suspension. This figure indicates that many larger agglomerates are collected at higher initial particle concentrations. (b) XRD results of the as-fabricated particles under the conditions in Fig. S1-a. This figure indicates that there were no changes in the intensity levels of Si and SiC, respectively. (c) The average size of the agglomerates increases from 0.34 µm to 0.8 µm as the concentration increased from 0.25 wt% to 1.0 wt%.


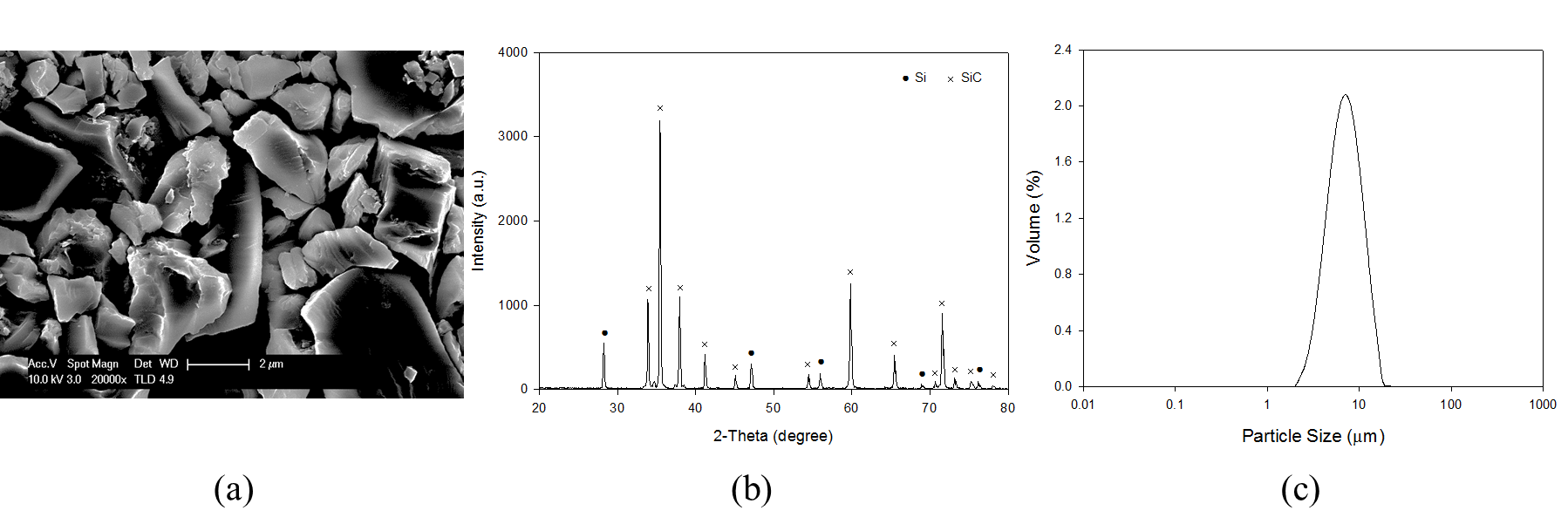


Figure S2. (a) SEM image, (b) XRD pattern and (c) particle size distribution of the residue in the revision after ultrasonic spray drying. After Si recovery, the residue in the reservoir of ultrasonic atomizer was mainly composed of SiC particles ranged from 2 to 20 μm. It is considered that the existence of remaining Si particles in the residue was due to strong agglomeration between Si and SiC particles.


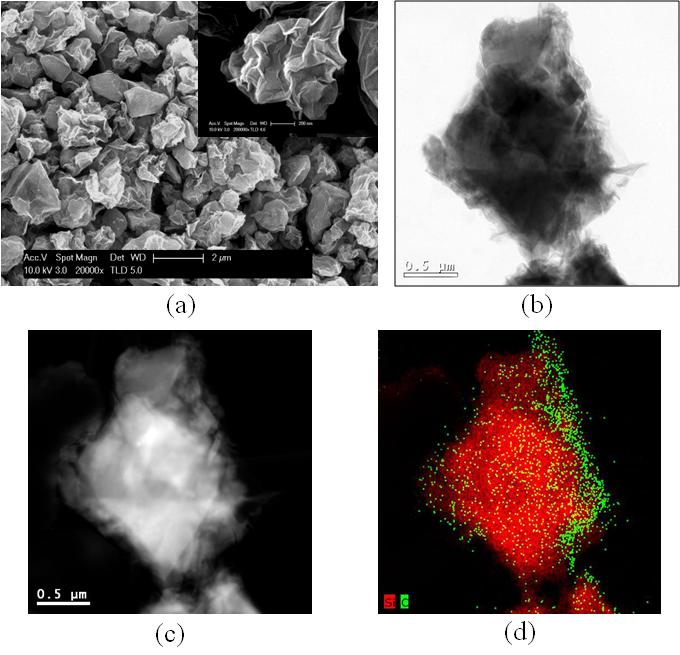


**Figure S3.** (a) SEM image of the as-fabricated GR-encapsulated Si composite with respect to the content of the particles. All of the as-fabricated composites had the appearance of a spherical ball and were uniform in size. (b) TEM analysis of the GR-encapsulated Si composite. (c) Z-contrast transmission mode of the as-fabricated composites. (d) EELS elemental mapping of the carbon and Si in the as-fabricated composites.
